# Cardiac Catheterization versus Echocardiography for Monitoring Pulmonary Pressure: A Prospective Study in Patients with Connective Tissue Disease-Associated Pulmonary Arterial Hypertension

**DOI:** 10.3390/diagnostics10010049

**Published:** 2020-01-19

**Authors:** Vasiliki Kalliopi Bournia, Iraklis Tsangaris, Loukianos Rallidis, Dimitrios Konstantonis, Frantzeska Frantzeskaki, Anastasia Anthi, Stylianos E. Orfanos, Eftychia Demerouti, Panagiotis Karyofillis, Vassilis Voudris, Katerina Laskari, Stylianos Panopoulos, Panayiotis G. Vlachoyiannopoulos, Petros P. Sfikakis

**Affiliations:** 1 First Department of Propaedeutic Internal Medicine and Joint Rheumatology Program, Medical School, National and Kapodistrian University of Athens, 157 72 Athens, Greece; lily_bournia@hotmail.com (V.K.B.); katerina_laskari@yahoo.gr (K.L.); sty.panopoulos@gmail.com (S.P.); 2 Pulmonary Hypertension Clinic, Attikon University General Hospital, Medical School, National and Kapodistrian University of Athens, 157 72 Athens, Greece; itsagkaris@med.uoa.gr (I.T.); lrallidis@gmail.com (L.R.); dkonstantonis@gmail.com (D.K.); ffrantzeska@gmail.com (F.F.); anastasia.anthi1@gmail.com (A.A.); stylianosorfanosuoa@gmail.com (S.E.O.); 3 Invasive Cardiology Department, Onassis Cardiac Surgery Center, 176 74 Kallithea, Greece; efidemer@otenet.gr (E.D.); pakar768@yahoo.gr (P.K.); vvoudris@gmail.com (V.V.); 4 Department of Pathophysiology and Joint Rheumatology Program, Medical School, National and Kapodistrian University of Athens, 157 72 Athens, Greece; pvlah@med.uoa.gr

**Keywords:** echocardiography, pulmonary arterial hypertension, systemic sclerosis, systemic lupus erythematosus, mixed connective tissue disease

## Abstract

Standard echocardiography is important for pulmonary arterial hypertension (PAH) screening in patients with connective tissue disease (CTD), but PAH diagnosis and monitoring require cardiac catheterization. Herein, using cardiac catheterization as reference, we tested the hypothesis that follow-up echocardiography is adequate for clinical decision-making in these patients. We prospectively studied 69 consecutive patients with CTD-associated PAH. Invasive baseline pulmonary artery systolic pressure (PASP) was 60.19 ± 16.33 mmHg (mean ± SD) and pulmonary vascular resistance (PVR) was 6.44 ± 2.95WU. All patients underwent hemodynamic and echocardiographic follow-up after 9.47 ± 7.29 months; 27 patients had a third follow-up after 17.2 ± 7.4 months from baseline. We examined whether clinically meaningful hemodynamic deterioration of follow-up catheterization-derived PASP (i.e., > 10% increase) could be predicted by simultaneous echocardiography. Echocardiography predicted hemodynamic PASP deterioration with 59% sensitivity, 85% specificity, and 63/83% positive/negative predictive value, respectively. In multivariate analysis, successful echocardiographic prediction correlated only with higher PVR in previous catheterization (*p* = 0.05, OR = 1.235). Notably, in patients having baseline PVR > 5.45 WU, echocardiography had both sensitivity and positive predictive values of 73%, and both specificity and negative predictive value of 91% for detecting hemodynamic PASP deterioration. In selected patients with CTD-PAH echocardiography can predict PASP deterioration with high specificity and negative predictive value. Additional prospective studies are needed to confirm that better patient selection can increase the ability of standard echocardiography to replace repeat catheterization.

## 1. Introduction

Pulmonary arterial hypertension (PAH), a severe complication of connective tissue disease (CTD), has been commonly described among systemic sclerosis (SSc) patients, with an estimated prevalence of 5–10% [1,2]. Less frequently, it can also be a manifestation of systemic lupus erythematosus (SLE), mixed connective tissue disease (MCTD), dermatomyositis, polymyositis, Sjögren’s syndrome and rheumatoid arthritis [3]. CTD-associated PAH usually carries a worse prognosis compared to idiopathic pulmonary arterial hypertension [4].

While standard echocardiography is an important non-invasive screening procedure for these patients, PAH diagnosis is only established by right heart catheterization (RHC) [5,6]. Follow-up of CTD patients with PAH includes standard echocardiography and RHC every 6–12 months, or 3–6 months after changes in therapy, according to the 2015 European Society of Cardiology/European Respiratory Society (ESC/ERS) guidelines [7]. Among the many parameters derived from standard echocardiography, Pulmonary Artery Systolic Pressure (PASP) remains the most commonly used. However, the exact role of echocardiographic parameters in the follow up of CTD-PAH patients and their subsequent correlation with hemodynamic and clinical prognostic factors has not yet been validated.

We undertook this study to test the hypothesis that follow-up standard echocardiography alone is adequate for clinical decision-making during monitoring of patients with connective tissue disease-associated PAH.

## 2. Materials and Methods

We prospectively studied 69 consecutive patients with CTD associated PAH, combining data from two independent cohorts of 45 and 24 patients, respectively. Diagnosis of PAH had been established by RHC; echocardiography was performed no more than 24 h before RHC. To avoid the effect of respiratory variation, both hemodynamic as well as echocardiographic parameters were measured at end-expiration [8]. Table 1 shows patient characteristics at baseline. Eighty-six percent of the patients were women. Mean ± SD age at baseline was 60.70 ± 11.90 years. In 58 cases, PAH was associated with SSc, in 5 cases with SLE and in 6 cases with MCTD. Baseline mean ± SD PASP was 60.19 ± 16.33 mmHg; baseline mean ± SD pulmonary vascular resistance (PVR) was 6.44 ± 2.95 Wood units. All 69 patients underwent repeat echocardiography and RHC after 9.47 ± 7.29 months; 27 patients had a third follow-up after 17.2 ± 7.4 months from baseline. Patient characteristics at follow-up are presented in Table 2. All participants gave written informed consent prior to enrolment. The study protocol was approved by the Attikon University General Hospital (approval number: ΕΒΔ410/17.9.2014, approval date: 17 September 2014) and the Onassis Cardiac Surgery Center (approval number: 618/23.03.2018, approval date: 23 March 2018) Ethics Committees.

For each patient, we measured differences in RHC-derived PASP and echocardiography-derived PASP between the 1st and the 2nd and the 2nd and 3rd assessment, thus creating in all 96 pairs of baseline and follow-up RHC-echocardiography evaluations. A clinically meaningful hemodynamic change of RHC-derived PASP and PVR from baseline to follow-up was defined as >10%. Likewise, a clinically meaningful change of echocardiography derived PASP was defined as >10%.

Low risk criteria for an adverse prognosis of PAH were recorded for all patients, both at baseline and at follow-up, including 6 min walking distance (6mWD), N-terminal Brain Natriuretic pro-peptide (NT-pro-BNP), Cardiac Index (CI), Right Atrial Pressure (RAP) and New York Heart Association (NYHA) functional class [9].

Bland-Altman analysis was performed, plotting the difference between change in RHC-derived PASP and change in echo-derived PASP, against the median of the two changes, from baseline to follow-up. McNemar’s test was used to determine if deterioration of RHC-derived PASP could be predicted by change in the corresponding echocardiography-derived PASP. Binary logistic regression analysis was performed to identify patient parameters that could affect the ability of echocardiography to predict deterioration in RHC-derived PASP at the follow-up assessment of CTD patients with established PAH. The sensitivity, specificity, positive predictive value and negative predictive value with which standard echocardiography could predict deterioration in RHC-derived PASP was calculated. SPSS statistical software package version 25 was used for all analyses. The level of statistical significance was set at *p* ≤ 0.05.

## 3. Results

Among 69 CTD patients with pulmonary arterial hypertension that underwent a second RHC after a mean of 9.47 ± 7.29 months, 30 patients improved their PASP, 26 remained stable, and 13 deteriorated. Likewise, of the 27 patients that underwent a third RHC, 7 improved, 4 remained stable and 16 deteriorated from second to third evaluation. Comparing PASP deterioration as measured by repeat RHC to that estimated by repeat echocardiography, we found that in 74 out of 96 patients’ retests (77%) the two methods coincided.

### 3.1. Sensitivity, Specificity, Positive Predictive Value, Negative Predictive Value

As shown in Table 3, both RHC and echocardiography detected ≥10% improvement or stability of PASP in 57 pairs and ≥10% deterioration of PASP in 17 pairs of baseline/follow-up comparisons. Using McNemar’s test for symmetry, we found no statistically significant difference in the ability of the two methods to detect ≥10% deterioration in PASP, when applied in the follow up of patients with CTD and established PAH (*p* = 0.629). In fact, echocardiography could predict deterioration of RHC-derived PASP at follow-up with 59% sensitivity, 85% specificity, 63% positive predictive value and 83% negative predictive value.

### 3.2. Bland Altman Analysis

**Figure 1:**
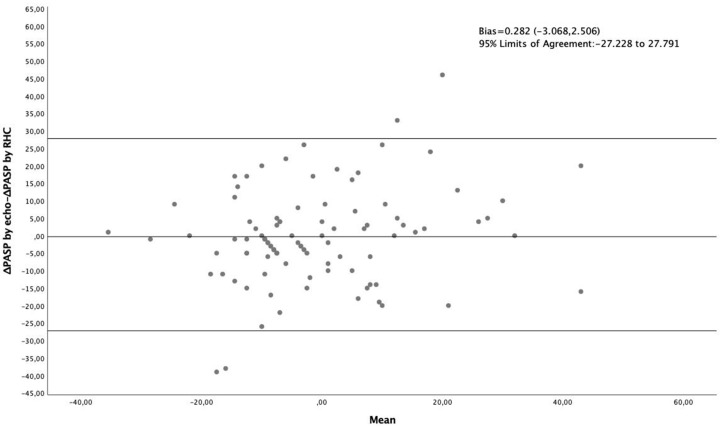
Bland-Altman analysis plotting the difference between change in RHC-derived PASP and change in echo-derived PASP against their average, in 69 CTD patients with established PAH (96 pairs of baseline–follow-up measurements.

Bland Altman analysis, plotting the difference between change in RHC-derived PASP and change in echo-derived PASP from baseline to follow up, against the mean of the two changes, showed a bias of 0.282 (−3.068, 2.506) with 95% Limits of Agreement: −27.228 to 27.791 (Figure 1). Linear Regression analysis showed the bias to be proportional (*p* = 0.039), increasing along with the mean.

### 3.3. Multivariate Binary Logistic Regression Analysis

Binary logistic regression analysis was used to identify possible correlations of different patient characteristics (age, gender, underlying CTD, time to follow-up, NT-pro-BNP, echo-derived PASP, RHC-derived PASP, PVR, CI and RAP < 8 mmHg at baseline, 6 mWD, NYHA functional class and number of low risk criteria for an adverse prognosis of PAH at baseline and at follow-up) with the successful or not prediction of PASP deterioration by follow-up echocardiography. Univariate binary logistic regression analysis revealed a marginal association of RHC-derived PVR at baseline (*p* = 0.052, OR = 1.225) and of echo-derived PASP at baseline (*p* = 0.077, OR = 1.027) with the ability of repeat echocardiography to predict PASP deterioration in repeat RHC. After correction for age and gender the association with RHC-derived PVR at baseline became significant (*p* = 0.050, OR = 1.235).

Using the Youden index in ROC curve analysis we defined a baseline PVR cutoff of 5.45 Wood Units for which prediction of PASP deterioration by repeat echocardiography was achieved with the maximum accuracy (sensitivity (70%) and specificity (62%)). In a selected population of patients with RHC-derived PVR >5.45 Wood Units at baseline (*n* = 58 pairs of baseline/follow-up comparisons) repeat echocardiography could predict PASP deterioration in RHC with 73% sensitivity, 91% specificity, 73% positive predictive value and 91% negative predictive value (Table 4).

## 4. Discussion

To the best of our knowledge this is the first study to assess the usefulness of standard echocardiography for the monitoring of CTD patients with established PAH using the deterioration of RHC-derived PASP as reference. Interestingly, there is an ongoing debate regarding the use of RHC [10] or non-invasive methods, such as echocardiography [11] in the follow-up evaluation of PAH, although to date, no study has directly compared the two methods. On the other hand, several previous studies have examined the correlation between echocardiographic and RHC measurements of PASP at PAH diagnosis with contradicting results.

The majority of these studies were not performed in CTD patients. In an interesting meta-analysis of 9 such studies, the correlation between echocardiography-derived PASP and RHC-derived PASP ranged from *r* = 0.65, *p* < 0.001 to *r* = 0.97, *p* < 0.001, while the pooled sensitivity and specificity of standard echocardiography in the diagnosis of pulmonary hypertension was 88% and 56%, respectively [12]. A more recent meta-analysis, including 2604 echocardiography-RHC pairings of PASP measurements for PAH diagnosis obtained from 32 studies, found an overall weighted correlation coefficient of 0.68 ± 0.19, with poorer correlations for right heart compared to left heart pathologies [13]. In addition, a meta-analysis of 29 studies comparing the accuracy of standard echocardiography to RHC for diagnosis of PAH, showed a sensitivity of 83% (95% CI: 73–90), a specificity of 72% (95% CI: 53–85) and a diagnostic odds ratio of 13 (95% CI: 5–13) [14]. Moreover, it revealed a correlation of 70% (95% CI: 0.67–0.73) between PASP estimated by standard echocardiography compared to that estimated by RHC. Finally, a multicenter, observational study including 2967 patients from the REVEAL registry (Registry to Evaluate Early and Long-Term PAH Disease Management), showed that although there is good correlation between RHC and echocardiographic measurements of PASP at baseline, there was little correlation between RHC and standard echocardiography at follow-up [15].

As far as CTD patients are concerned, a small study by Denton et al. comparing echocardiographic assessment with RHC for PAH initial diagnosis in 33 SSc patients showed that echocardiography identified PAH with a sensitivity of 90% and a specificity of 75% [16]. Another study that compared RHC to non-invasive methods for the diagnosis of PAH reported that standard echocardiography classified 38/49 SSc patients correctly with a sensitivity of 58% and a specificity of 96%. Moreover, magnetic resonance imaging measurement of pulmonary artery diameter had a sensitivity of 68% and a specificity of 71% and pulmonary function tests had a sensitivity of 71% and a specificity of 72% [17].

According to the 2015 European Society of Cardiology/European Respiratory Society (ESC/ERS) guidelines, RHC should be always considered for the follow up of CTD patients with PAH 3–6 months after changes in therapy, or in patients who experience clinical deterioration. Additionally, at some centers, RHC is routinely performed at annual or semi-annual intervals during follow-up [7]. Moreover, PAH patients should undergo a multidimensional periodic risk evaluation, which provides information on prognosis and guidance to therapeutic choices. According to ECS/ERS guidelines, three risk categories (low, intermediate and high) are identified, based on clinical, imaging, hemodynamic and exercise parameters [7]. Importantly, several hemodynamic variables provided by RHC, including right atrial pressure (RAP), pulmonary arterial compliance, pulmonary vascular resistance (PVR), cardiac index (CI) and stroke volume index (SVI) are extremely useful in patient assessment, since they are indicative of right ventricle function and have been associated with the survival of PAH [18] and SSc-PAH patients [19]. The French PAH registry validated the significance of several invasive and non-invasive low risk criteria, for the risk assessment in idiopathic, heritable and drug-induced PAH [9].

Recently, the prognostic value of these parameters was studied in CTD-PAH patients. Weatherald et al. assessed the role of follow up hemodynamic parameters and low risk criteria as prognostic factors in SSc-PAH patients [19]. Moreover, Mercurio et al. demonstrated the validity of ECS/ERS risk assessment on predicting prognosis of SSc-PAH patients [20]. However, the exact role of echocardiographic parameters in the follow up of CTD-PAH patients and their subsequent correlation with hemodynamic and clinical prognostic factors has not been validated.

In the present study, we first showed that, in CTD patients with established PAH, the echocardiographic estimation of PASP deterioration at follow-up coincided with the respective hemodynamic measurement in 77% of baseline/follow up comparisons. Echocardiography could predict PASP deterioration with a low sensitivity (59%) and positive predictive value (63%), but with an acceptable specificity (85%) and negative predictive value (83%). In addition, in the Bland-Altman analysis, the bias was small (−0.282 (−3.068, 2.506)) but proportional to the mean and the 95% limits of agreement were quite wide, meaning that standard echocardiography cannot readily substitute for RHC in the assessment of change of PASP.

However, and more importantly, multivariate binary logistic regression analysis showed that baseline RHC-derived PVR is significantly associated with the accuracy of repeat echocardiography in predicting PASP deterioration. Thus, in the selected subgroup of patients with baseline RHC-derived PVR >5.45 Wood Units, repeat echocardiography could identify PASP deterioration with a low sensitivity (73%) and positive predictive value (73%), but with a relatively high specificity (91%) and negative predictive value (91%). Overall, in this patient subgroup, standard echocardiography performs much better compared to the general population of CTD related PAH patients, as it correctly rules out PASP deterioration in 91% of patients.

An interesting and rather unexpected finding in our study was that baseline hemodynamic indices were extremely similar between patients in NYHA functional class II and III and only seemed to deteriorate in patients belonging to functional class IV (Table 2). Patient categorization into different functional classes was performed by experienced clinicians, therefore the risk of bias or misclassification was minimal. As a result, we believe that other reasons relating to the underlying CTD could have also accounted for dyspnea and low exercise tolerance in our patient group, for example the coexistence of interstitial lung disease.

An important strength of our study is the sequential echocardiographic and hemodynamic evaluation of CTD-PAH patients on two or three visits, and the respective recording of risk stratification parameters. Regarding the prognosis of those patients, there is no consensus on which echocardiographic parameter could more accurately predict the outcome. Impaired right ventricular function is associated with mortality, and improvement of RV function might be a significant aim of treatment [21]. Therefore, in addition to the small size, a limitation of our study might be that we did not record other markers of RV dysfunction [22]. Further limitations are the lack of quality assessment of Doppler curve, by using a predefined score, as well as the noninvasive estimation of right atrial pressure from the combination of the inferior vena cava diameter and respiratory collapse [23]. Although recommended by the European and Canadian guidelines, the estimation of right atrial pressure with this method has been criticized for its accuracy [24] and alternative strategies have been proposed focusing on non-invasive measurement instead of estimation [25]. Finally, and although the time interval between hemodynamic and echocardiographic measurements was less than 24 h, we cannot exclude a time variation factor as has been previously demonstrated in this setting [24].

## 5. Conclusions

To conclude, these results suggest that in selected patients with connective tissue disease-associated PAH non-invasive monitoring can predict PASP deterioration with a high specificity and negative predictive value. Additional prospective studies are needed to define whether better selection of patients could increase the ability of standard echocardiography to guide treatment decisions in patients who are in need for frequently repeated cardiac catheterizations. Reducing the necessity for routine follow-up RHC even in a small subset of patients with CTD-associated PAH could be important, given the invasive nature, high cost and low availability of this procedure [11].

## Figures and Tables

**Table 1:** Baseline characteristics of connective tissue disease patients in the two cohorts combined.

		N (%)	Age (Years)	Echo-Derived PASP (mmHg)	RHC-Derived PASP (mmHg)	PVR (WU)	Cardiac Index	RAP (mmHg)	NT-pro-BNP (pg/mL)	6mWD (m)
Underlying disease	SSc	58 (84)	61.45 ± 11.68	65.72 ± 17.01	59.02 ± 16.23	6.41 ± 2.96 (*n* = 55)	2.50 ± 0.63	6.62 ± 3.52	1848.69 ± 2643.41 (*n* = 45)	357.74 ± 124.69 (*n* = 43)
	MCTD	6 (9)	63.17 ± 8.75	61.67 ± 27.66	60.50 ± 13.66	6.35 ± 3.39	2.40 ± 2.4	8.17 ± 3.54	1493.67 ± 1540.89 (*n* = 3)	378.0 ± 75.78
	SLE	5 (7)	49.00 ± 13.3	64.20 ± 19.38	73.40 ± 17.57	7.05 ± 2.79 (*n* = 4)	2.42 ± 0.10	9.0 ± 4.53	4142.75 ± 5678.68 (*n* = 4)	290.0 ± 262.11 (*n* = 3)
Gender	female	59 (86)	61.64 ± 11.95	63.58 ± 17.82	59.31 ± 16.81	6.42 ± 3.12 (*n* = 57)	2.51 ± 0.58	6.82 ± 3.71	2129.72 ± 3053.44 (*n* = 46)	351.94 ± 132.31 (*n* = 48)
	male	10 (14)	55.10 ± 10.47	75.2 ± 16.14	65.4 ± 12.57	6.55 ± 1.24 (*n* = 8)	2.32 ± 0.59	7.6 ± 2.99	1046.0 ± 814.72 (*n* = 4)	407.0 ± 19.90 (*n* = 4)
NYHA stage	I	0	-	-	-	-	-	-	-	-
	II	22 (32)	56.91 ± 12.01	55.45 ± 13.43 *	56.77 ± 13.49	5.55 ± 1.7	2.56 ± 0.33	6.18 ± 2.36	723.76 ± 1013.77 (*n* = 17)	442.6 ± 69.80 ** **(*n* = 20)**
	III	37 (54)	62.38 ± 11.85	65.57 ± 16.94 *	57.72 ± 16.31	5.95 ± 2.79 (*n* = 34)	2.57 ± 0.66	6.49 ± 3.62	1657.48 ± 2004.25 (*n* = 27)	348.62 ± 65.98 ** **(*n* = 26)**
	IV	10 (14)	62.80 ± 10.94	85.7 ± 12.92 *	**76.8 ± 12.88 ^#^**	**10.49 ± 2.89 ^#^** **(*n* = 9)**	**2.00 ± 0.51 ^#^**	**10.2 ± 4.37 ^#^**	**5897.37 ± 4717.60 ^#^** **(*n* = 8)**	100.83 ± 135.44 ** **(*n* = 6)**
Total cohort		69 (100)	60.70 ± 11.90	65.26 ± 17.95	60.19 ± 16.33	6.44 ± 2.95	2.48 ± 0.59	6.93 ± 3.61	2004.67 ± 2900.68	356.17 ± 127.96

Continuous values are presented as mean ± SD. (SSc: Systemic Sclerosis, MCTD: Mixed Connective Tissue Disease, SLE: Systemic Lupus Erythematosus, NYHA: New York Heart Association, PASP: Pulmonary Artery Systolic Pressure, RHC: Right Heart Catheterization, PVR: Pulmonary Artery Resistance, RAP: Right Atrial Pressure, NT-pro-BNP: N-terminal pro Brain Natriuretic Peptide, 6mWD: 6 min Walking Distance).* *p* = 0.052 for NYHAII vs NYHA III, *p* < 0.05 for NYHA II vs NYHA IV and NYHA III vs NYHA IV; ^#^ *p* < 0.05 for NYHA II vs NYHA IV and NYHA III vs NYHA IV; ** *p* < 0.001 for all comparisons.

**Table 2:** Follow-up characteristics of connective tissue disease patients in the two cohorts combined.

		1st Follow-up	2nd Follow-up
N (%)		69 (100%)	27 (39%)
Age (years)		61.35 ± 12.25	61.80 ± 11.85
Female gender (%)		59 (86)	24 (89)
Underlying disease	SSc	58 (84)	24 (89)
	MCTD	6 (9)	1 (4)
	SLE	5 (7)	2 (7)
Time to follow-up (months)		9.47 ± 7.29	8.29 ± 4.36
NYHA stage (%)	I	2 (3)	-
	II	32 (46)	14 (52)
	III	33 (48)	13 (48)
	IV	2 (3)	-
Echo-derived PASP (mmHg)		62.65 ± 20.30	68.70 ± 26.90
RHC-derived PASP (mmHg)		56.65 ± 16.5	65.30 ± 18.63
PVR (wood units)		6.02 ± 3.04	7.34 ± 3.15
Cardiac Index		2.68 ± 0.59	2.51 ± 0.37
RAP (mmHg)		7.33 ± 4.3	7.26 ± 4.06
NT-pro-BNP (pg/mL)		1197.89 ± 1507.77 (*n* = 54)	1356.4 ± 1300.64 (*n* = 20)
6mWD (m)		389.20 ± 83.94 (*n* = 46)	412.0 ± 79.28 (*n* = 16)

Continuous values are presented as mean ± SD. (SSc: Systemic Sclerosis, MCTD: Mixed Connective Tissue Disease, SLE: Systemic Lupus Erythematosus, NYHA: New York Heart Association, PASP: Pulmonary Artery Systolic Pressure, RHC: Right Heart Catheterization, PVR: Pulmonary Artery Resistance, RAP: Right Atrial Pressure, NT-pro-BNP: N-terminal pro Brain Natriuretic Peptide, 6mWD: 6 min Walking Distance).

**Table 3:** Deterioration in echo-derived PASP at follow-up predicts deterioration in repeat RHC-derived PASP with 59% sensitivity, 85% specificity, 63% positive predictive value and 83% negative predictive value. (PASP: Pulmonary Artery Systolic Pressure, RHC: Right Heart Catheterization).

		RHC-Derived PASP		
		Deteriorated ≥ 10%	Stable or Improved ≥ 10%	Total
Echo-derived PASP	Deteriorated ≥ 10%	17	10	27
	stable or improved ≥ 10%	12	57	69
	total	29	67	96

McNemar’s test, *p* = 0.629.

**Table 4:** In a selected subgroup of patients with baseline echo-derived PVR > 5.45 Wood Units, deterioration in echo-derived PASP at follow-up predicts deterioration in repeat RHC-derived with 73% sensitivity, 91% specificity, 73% positive predictive value and 91% negative predictive value. (PASP: Pulmonary Artery Systolic Pressure, RHC: Right Heart Catheterization).

		RHC-Derived PASP		
		Deteriorated ≥ 10%	Stable or Improved ≥ 10%	Total
Echo-derived PASP	Deteriorated ≥ 10%	11	4	15
	stable or improved ≥ 10%	4	39	43
	total	15	43	58

McNemar’s test, *p* = 0.063.
